# Prestin and the good vibrations

**DOI:** 10.1042/BCJ20160015

**Published:** 2016-07-28

**Authors:** Anna Sofia Birke, Arnaud Javelle

**Affiliations:** * University of Strathclyde, Strathclyde Institute of Pharmacy and Biomedical Sciences, 161 Cathedral Street, Glasgow, G40RE, United Kingdom

**Keywords:** cochlea, hearing system, outer hair cell, prestin, SLC26A5, STAS domain

## Abstract

In a recent paper published in the *Biochemical Journal*, Lolli et al. presented evidence that the C-terminal STAS (sulfate transporter and anti-sigma factor antagonist) domain of the motor protein prestin possesses an anion-binding site. This discovery might shed light on an aspect of the function of this mysterious and fascinating protein that is crucial for the human hearing system.

Prestin [SLC26A5 (solute carrier 26A5)] belongs to the ubiquitous SLC26/SulP (sulfate permease) family of anion exchangers and is one of ten SLC26A transporters that have been identified in animals, most of which are known to transport anions across membranes either in an electroneutral or electrogenic manner [1]. In humans, genes encoding four members of the SCL26A protein family have been identified as disease genes: mutations in the genes encoding SLC26A2/DTD, SLC26A3/DRA, SLC26A4/pendrin and SLC26A5/prestin are associated with diastrophic dysplasia, congenital chloride-losing diarrhoea, Pendred syndrome and human deafness, respectively [1]. Although prestin is a member of a mammalian anion-exchanger family, it apparently has a very different function in cochlear OHCs (outer hair cells), where it is densely packed in the basolateral membrane. OHCs in mammals have the ability to alter their cell length in response to changes in membrane potential triggered by incoming sound waves. This form of voltage-dependent cell movement, known as electromotility, is rendered possible by prestin. Therefore prestin is often referred to as an OHC motor protein. OHC electromotility is part of the mechanical sound amplification system responsible for increased hearing sensitivity and frequency selectivity in mammals. In contrast with prestin-based electromotiliy in mammals, non-mammalian prestin orthologues display more ‘traditional’ anion-transporting activity [2,3]. However outstanding mammalian prestin might be, it shares its topology and a highly conserved intracellular C-terminal domain called STAS (sulfate transporter and anti-sigma antagonist) domain with other members of the SLC26 family [2,4]. Despite decades of effort in research to solve prestin's structure and to fully understand the underlying mechanism of action, neither mission has yet been accomplished. However, in early 2016, Lolli et al. [5] presented results in the *Biochemical Journal* that suggest the mammalian prestin STAS domain possesses an anion-binding site, to which the physiologically relevant Cl^−^ ion binds. The idea that intracellular Cl^−^ ions might act as an extrinsic voltage sensor for prestin makes this discovery all the more exciting [6].

Electromotility of cochlear OHCs, which is their ability to elongate or contract actively and quickly (>20 kHz in humans) in response to changes in membrane potential, was first observed in 1985 [7]. Over the course of the following 15 years, the driving force underlying this form of voltage-dependent cellular response would remain unknown. With guinea pig OHCs, the changes in cell length could be observed after as short a time as 120 μs post-stimulation, which is far more rapid than any actin–myosin-based cell motility functions [8]. This observation suggested that the mechanism underlying electromotility was different from any molecular motor that was known at the time. Furthermore, the mechanism was also shown to work independently of ATP [9], and so it became increasingly apparent that OHC electromotility is based on a novel type of force-generating mechanism. Additional clues as to what might be driving electromotility included the description of IMPs (intermembrane particles) in the 1970s [10], as well as the finding that OHCs whose cell content had been removed via tryptic digest were still able to perform electromotile movements [11]. These results provided enough evidence to come to the conclusion that the mysterious driving force of OHC electromotility should manifest itself in a membrane-associated protein that is located in the lateral membrane wall of OHCs and that has the ability to undergo rapid conformational changes in a voltage-dependent manner. Fast-forward to the turn of the millennium and Zheng et al. [12] solved that part of the puzzle with the discovery of prestin, which is named after its most outstanding feature: the speed at which it operates (musical notation *presto*=fast).

Prestin is the fifth mammalian member of the SLC26A proteins, which in turn belong to the large family of SLC26/SulP anion-transporter-related proteins [2]. Members of the family are widely distributed among animals, bacteria, plants and fungi. With 700–1000 amino acids, SLC26/SulP proteins are relatively large membrane proteins. Prestin itself is composed of 744 amino acids. Members of the family appear to have a very similar topology with 10 to 14 TM (transmembrane) domains that contain a conserved cytoplasmic C-terminal motif, named the STAS domain. Although the function of the prestin STAS domain has not yet become quite clear, it was thought to be important for targeting to the membrane in many SLC26/SulP proteins [1,12,13]. However, more recent work using bacterial SLC26A transporters whose STAS domains have been replaced with the GFP motif have shown that the STAS domain does not play a direct role in protein targeting, but rather in protein stabilization and certainly protein function [14].

Despite belonging to a family of anion-transporter-related proteins, mammalian prestin is not an anion transporter as such. Instead, it is the protein driving electromotiliy of OHCs by undergoing incredibly rapid conformational changes. These generate a force great enough to result in alteration of OHC length. The resulting mechanical amplification of sound waves is a mechanism unique to mammals. OHCs and IHCs (inner hair cells) are two distinct types of mechanosensory cells found in the organ of Corti, where IHCs function as primary auditory signal receivers. Sound amplification by OHCs occurs in a cycle-by-cycle manner and relies on the ability of prestin to sense changes in membrane potential, either depolarization or hyperpolarization. In response to that, it must be able to change its conformation very quickly, causing the cell to either contract or elongate. Thus, presumably, prestin has at least two functional domains, namely the voltage-sensor and the actuator.

As mentioned above, prestin in mammals and non-mammalian animals does not have the same function despite amino acid similarity. Although only prestin in mammals acts as a molecular motor that drives electromotility in OHCs, the non-mammalian orthologue functions as a divalent/chloride anion exchanger [3]. Interestingly, the construction of a synthetic prestin (SynPres), composed of zebrafish prestin for most parts and some TM domains from rat prestin, appears to have the best of both worlds. SynPres has anion-exchanging abilities in the way that zebrafish prestin does and SynPres-transfected cells feature electromotility. The generation and assessment of SynPres function delivered results indicating that the actuator domain, that is the protein region responsible for inducing fast conformational changes, might be located in the TM part of prestin [15]. But how does prestin ‘measure’ changes in membrane potential? It has been proposed that binding of Cl^−^ from the cytoplasmic side may act as an extrinsic voltage sensor since the removal of intracellular Cl^−^ resulted in the absence of electromotility of rat OHCs [6].

It was not until 2016, when Lolli et al. [5] published their paper ‘The STAS domain of mammalian SLC26A5 prestin harbours an anion-binding site’, that the idea of intracellular Cl^−^ anions potentially playing a part in voltage sensing was confirmed by structural analysis of the rat prestin (rPres) STAS domain. Lolli et al. [5] examined several versions of both rat and chicken STAS domains, of which the former was bound to a range of different anions, among them Cl^−^, in order to assess conformational changes that may occur within the STAS domains. To obtain crystal structures of the STAS domain, it was necessary to delete a region of the domain called the variable loop, which has a length of 74 amino acids in rPres STAS and 83 amino acids in the chicken prestin (cPres) STAS domain. By comparing the structures of rPres STAS in the presence of Cl^−^ in the alleged anion-binding site and in the absence thereof, it does not appear that the crystallized part of the protein undergoes any major conformational rearrangements as a result of Cl^−^ binding. This indicates that the anion-binding site maintains its conformation throughout the process of anions binding and dissociating, which underpins the concept of the binding site to be pre-formed and ready to accommodate anions that could actuate prestin function. In fact, all other anions, besides Cl^−^, that were also shown to bind to the crystallized rPres STAS domain trigger prestin activity, which indicates a specificity for these anions. In addition to solving the structure of the rPres STAS domain, Lolli et al. [5] obtained the crystal structure of the cPres STAS domain. They found that the anion-binding site present in the mammalian STAS domain was, indeed, absent from the bird version of the protein, which suggests that anion binding to a pre-formed cavity is likely to be a unique property of the mammalian orthologue and possibly linked to the difference in biological function. The authors propose that the anion-binding site in the mammalian prestin STAS domain might function as a low-affinity ‘ready-to-use’ reservoir of actuator anions [5]. Clearly, the crystal structures obtained suggest that no conformational rearrangements in the STAS domain occur upon Cl^−^ binding. However, this may be a crystallization artefact or the result of the missing variable loop, as well as the absence of the rest of the prestin protein [5].

Despite the progress brought about by revelations such as the identification of the C-terminal anion-binding site within the STAS domain, unanswered questions remain. Although Cl^−^ is clearly a key component of prestin's voltage-sensing ability, it poses a chicken-and-egg problem. Does the binding of Cl^−^ anions to prestin act as an extrinsic voltage sensor or is it the consequence of a preliminary intrinsic voltage-sensing mechanism? Although anions might be binding to the site in the STAS domain, the mystery of its actual function and how it fits in with the rest of the prestin protein has not yet been solved. Recently, the crystal structure of a SLC26 protein from *Deinococcus geothermalis*, SLC26Dg, was obtained and published as the first ever complete structure of a SLC26 protein [16]. Unfortunately, the structure gives no clue about the functional relationship of the TM domain and the STAS domain within SLC26Dg [16]. Besides the questions concerning the interaction between the TM domain and the STAS domain and the general function of the latter, examination of the functional actuator domain, once identified, should be particularly interesting from an evolutionary point of view. How and why did prestin evolve in a way that it lost its anion-exchanging capabilities and simultaneously gained the power to generate electromotility-driving forces in mammalian OHCs? Last, but not least, how is prestin able to function at such high speed; so very *presto*?

## Abbreviations

cPres: chicken prestin
IHC: inner hair cell
OHC: outer hair cell
rPres: rat prestin
SLC26: solute carrier 26
STAS: sulfate transporter and anti-sigma antagonist
SulP: sulfate permease
SynPres: synthetic prestin
TM: transmembrane
